# Upright positioning in pediatric radiotherapy: rationale, safety considerations and a research roadmap^☆^

**DOI:** 10.1016/j.tipsro.2026.100432

**Published:** 2026-09-03

**Authors:** Lorenzo Placidi, Kathryn Yip, Yat Man Tsang, Samuel Rodriguez, Lawrie Skinner, Tracy Underwood, Niek Schreuder

**Affiliations:** a Diagnostic Imaging and Oncological Radiotherapy Department, Fondazione Policlinico Universitario A. Gemelli, IRCCS, Rome, Italy; b Leo Cancer Care, Crawley, United Kingdom; c Radiation Medicine Program, Princess Margaret Cancer Centre, Toronto, Canada; d Department of Anesthesia, Critical Care, and Pain Medicine, Stanford University School of Medicine, Stanford, California, USA; e Stanford University, California, USA; f Leo Cancer Care, Middleton, USA

**Keywords:** Upright radiotherapy, Pediatric radiation therapy, Anesthesia avoidance, Person-centered care

## Abstract

Upright positioning is re-emerging as a potential strategy to improve the pediatric radiotherapy experience, particularly for selected children in whom distress, limited cooperation, or repeated general anesthesia represent major barriers to treatment. This practice development report, produced by the Upright Radiotherapy Pediatric Task Group — an international, multiprofessional group bringing together radiation oncology, medical physics, radiation therapy, pediatric anesthesia and industry expertise — reviews the historical rationale, contemporary technological developments, and clinical requirements for evaluating upright pediatric radiotherapy. The considerations discussed apply to both photon and proton delivery, although the current economic and dosimetric drivers are strongest for gantry-less proton systems. The concept is supported by historical experience with seated treatment techniques, recent advances in upright imaging and gantry-less delivery systems, and broader pediatric evidence suggesting that seated positioning may improve procedural tolerability. However, pediatric-specific evidence remains limited, and upright treatment should not be regarded as a universal alternative to conventional supine workflows.

Safe translation requires a deliberately cautious framework. Key considerations include age-appropriate immobilization, upright imaging and treatment-planning validation, both day-to-day reproducibility and within-fraction stability of setup, gravity-related anatomical changes, audiovisual distraction, and integration of child-centered preparation strategies. For sedated or anesthetized children, upright positioning introduces specific safety requirements related to airway visibility and access, hemodynamic monitoring, patient support, emergency release, rapid transition to a rescue position, and sufficient free space around the patient for the anesthesia team to intervene. These constraints should be treated as primary design and commissioning requirements rather than secondary workflow adaptations.

Near-term research priorities include phantom and anatomical validation studies, assessment of imaging and dosimetric accuracy, emergency workflow testing, and prospective pilot studies evaluating setup reproducibility and within-fraction stability, treatment duration, anesthesia utilization, acute safety, and patient- and caregiver-reported experience. If developed within a rigorous multidisciplinary safety and evidence framework, upright pediatric radiotherapy may become a clinically valuable option for selected children, complementing rather than replacing established supine treatment approaches.

## Highlights

• Upright radiotherapy may reduce distress in selected pediatric patients.
• Safety-led design must prioritize airway access and emergency release.
• Audiovisual support is central to awake upright pediatric treatment.
• Prospective studies should assess setup, anesthesia use and experience.
• Upright treatment should be evaluated as a child-centred extension of care.

## Introduction

Pediatric radiation therapy is one of the most complex areas in modern oncology, requiring precise treatment while accommodating the child's developmental stage, behavior, and emotional needs [1]. Because children spend most waking hours upright, treatment in this posture may be more intuitive and tolerable than lying flat awake. Upright positioning therefore represents a clinically plausible strategy for improving cooperation and reducing reliance on general anesthesia in selected patients, a premise that provides the clinical motivation for this report.

Renewed interest in upright radiotherapy has been building over the past decade, driven by converging technological and economic pressures within radiation oncology [2], [3]. Conventional proton therapy, the modality with the strongest dosimetric rationale for pediatric malignancy given its capacity to reduce dose to developing normal tissues, depends on purpose-built facilities costing between 30 and 50 million US dollars per treatment room, restricting access to a small number of specialist centres globally [4]. Gantry-less upright systems, in which the beam source is fixed and the patient rotates on a robotic chair, require primary shielding in only one direction, enabling installation within existing linear accelerator vaults at substantially reduced cost and with a smaller structural footprint [5]. For pediatric radiotherapy, where the clinical case for proton therapy is strongest and access is most constrained by cost and geography, this development warrants close attention. The rationale is not, however, confined to protons. Upright photon treatment has the longer historical track record (Section 2) and can be delivered either with conventional linear accelerators combined with an upright positioning system or with fixed-beam photon geometries. Except where a statement is explicitly modality-specific, the clinical requirements, safety considerations and research priorities set out in this report apply equally to upright photon and upright proton radiotherapy.

A central challenge in pediatric radiotherapy is the frequent need for anesthesia to ensure immobility and treatment accuracy [6]. Repeated anesthesia is not without risk; it prolongs treatment slots, places sustained pressure on anesthesia staffing, and adds logistical and emotional burden on families across multiweek treatment courses [6]. Strategies that improve a child's comfort and cooperation in the treatment room therefore carry value beyond patient experience. If upright positioning makes treatment more acceptable for some children, it may help reduce anesthesia use within the pediatric population.

Upright anesthesia is already practiced in other clinical settings, including dental, neurosurgical, and shoulder procedures, indicating that the posture itself is not inherently prohibitive [7]. In parallel, upright radiotherapy has historical precedent, particularly in Hodgkin lymphoma and thoracic treatment techniques, where it was used to improve immobilization and, in some cases, reduce radiation exposure to normal tissues [8], [9], [10], [11], [12], [13], [14], [15], [16].

This practice development report reviews the rationale for upright pediatric radiotherapy and proposes a framework for its safe clinical translation. It summarizes historical and contemporary evidence, anesthesia burden, safety considerations, audiovisual support, clinical and device requirements, workflow implications, and research priorities. The goal is not immediate widespread adoption, but to define the conditions of responsible evaluation and safe implementation.

This report originates from the Upright Radiotherapy Pediatric Task Group, an international and multiprofessional working group established in July 2025 to examine whether, and under what conditions, upright positioning could be applied safely to children. The group was established by Niek Schreuder of Leo Cancer Care and currently comprises 15 members from six institutions across Canada, Italy, the United Kingdom, and the United States. Its multidisciplinary membership includes radiation oncologists, medical physicists, radiation therapists, pediatric anesthesiologists, and industry engineers. Its terms of reference are to: (i) review the historical and contemporary evidence base for upright radiotherapy relevant to children; (ii) identify the safety-critical requirements for awake and anesthetized pediatric upright treatment; (iii) define minimum clinical and technical design requirements for upright systems intended for pediatric use; and (iv) propose a staged research and implementation roadmap with explicit evidence thresholds. The group meets every two months by teleconference. It operates without a formal sponsor and has received no dedicated financial, administrative, or in-kind support. Membership is by invitation and open on request. The group has no mandate to issue guidelines, and this report is a practice development statement reflecting the consensus view of its authors rather than a clinical guideline.

Three authors (K.Y., T.U., N.S.) are employed by Leo Cancer Care, a company that develops upright patient positioning and imaging equipment. Because the subject of this report is a technology to which their employer is commercially exposed, the following measures were adopted during its preparation. First, the scope, structure and principal conclusions were defined and agreed by the authors without a commercial affiliation before drafting began. Second, no passage making a recommendation about clinical adoption, safety requirements, evidence thresholds or research priorities was written or finally edited by an author with a commercial interest; the industry-affiliated authors contributed technical content on device capabilities and engineering constraints, which was subsequently verified against published sources by the non-affiliated authors. Third, the report deliberately avoids naming, comparing or endorsing specific commercial systems, and no performance claim attributable to a single vendor is made; where a commercial system is referred to, this is done exclusively through peer-reviewed publications. Fourth, the manuscript was reviewed in full by all authors, and the corresponding author (L.P.), who has no commercial affiliation, retained final editorial control over content and wording. Finally, the report makes no claim that upright treatment is superior to supine treatment for any pediatric indication; its central recommendation is that the approach requires prospective, safety-led evaluation before clinical adoption.

## Historical foundations and clinical evidence

Upright positioning has historical precedent in pediatric and adolescent radiotherapy, particularly in Hodgkin lymphoma treatment during the 1950s and 1960s [8], [9]. Early systems incorporated back, arm, head, and foot supports to improve immobilization and comfort during extended-field treatment [5]. Subsequent developments in treatment chairs and mantle-field techniques aimed to improve support, reproducibility, and normal tissue sparing [10], [11], [12], [13], [14].

Important technical advances followed in the 1980s with the introduction of thermoplastic immobilization masks and isocentric chair systems [15], [16]. Verhey et al. reported mean intra-fraction motion of 0.8 mm in pediatric head and neck radiotherapy using upright thermoplastic immobilization, showing that upright positioning could achieve geometric precision comparable with conventional approaches [15]. Isocentric chair systems also allowed treatment field changes without repeated patient repositioning, supporting efficient setup and delivery [16]. In the 2020s, multiple commercial vertical CT scanners and rotating chair systems have entered the market, contributing to renewed interest in upright radiotherapy [3]. Contemporary adult experience is now accumulating. A prospective clinical trial of gantry-less, upright, image-guided adaptive proton therapy for locally recurrent head and neck and brain malignancies reported feasible delivery with acceptable acute toxicity in a planned interim analysis of 20 patients [17]. A comparative image-guidance analysis of adult head and neck patients treated in both postures found residual setup errors in the upright position to be of the same order as those obtained supine, with median translational residuals below 1 mm in both cases [18]. Anatomical studies of the thorax further indicate that the transition from supine to upright can increase the separation between target volumes and some organs at risk, providing a physical rationale for potential dosimetric gains that nonetheless requires site-specific confirmation [19].

The first pediatric clinical experience with a modern upright system has recently been reported. A case report from Hadassah Medical Center in Israel provides the first clinical proof-of-concept for upright pediatric proton craniospinal irradiation (CSI) under anesthesia. Blumenfeld et al. treated a 4-year-old child with central nervous system (CNS) relapse of neuroblastoma using a gantry-less proton system with a fixed horizontal beamline, 6D robotic chair, upright CT-based image guidance, and orthogonal kilovoltage (kV) verification. The patient received 18.0 Gy relative biological effectiveness (RBE) in 12 fractions to the craniospinal axis, with a simultaneous integrated boost to 21.6 Gy (RBE) and a sequential boost to 30.6 Gy (RBE) to the resection cavity. Daily monitored anesthesia care was delivered without intubation, maintaining spontaneous respiration and airway access. Customized immobilization and daily upright CT registration enabled reproducible treatment, which was completed without interruption and without grade ≥ 2 acute toxicity. Although limited to a single case, this report supports the feasibility of upright proton CSI in anesthetized pediatric patients [20].

Evidence from broader pediatric care also supports the possible behavioral advantages of upright positioning. Children undergoing other procedures, such as vaccinations, often show less fear and distress when positioned upright rather than lying flat [21], [22], [23]. Seated positioning can support eye contact with caregivers and staff, provide a greater sense of control, and improve procedural tolerability. These findings are not radiotherapy-specific, but are relevant because pediatric radiotherapy depends on repeated daily cooperation, and increasing patient compliance is likely to reduce the use of general anesthesia.

A recent systematic review of anesthesia in pediatric radiotherapy further confirmed that dependence on general anesthesia remains substantial across centers globally, underscoring the clinical urgency of alternative approaches [24]. However, contemporary pediatric evidence for upright radiotherapy remains limited to a single reported case [20], and most recent technological development in upright treatment has focused on adults [2], [3]. Prospective pediatric studies using modern immobilization and image guidance are therefore needed to define present-day safety, reproducibility, stability, and clinical value.

## Demographics and anesthesia burden in pediatric radiotherapy

Pediatric radiotherapy presents distinct operational challenges, because treatment success often depends on a child's ability to remain still, manage anxiety, and cooperate with immobilization and image guidance. As a result, general anesthesia and deep sedation remain common.

Globally, approximately 300,000 children are diagnosed with cancer each year, and an estimated 40–50% require radiotherapy, most commonly for CNS tumors, leukemia and lymphoma, sarcomas, and neuroblastoma [25]. Brain and neuro-axis tumors account for a large proportion of indications and often require technically demanding regimens such as CSI. These treatments often require multiple radiation fields, are lengthy, highly conformal, and sensitive to setup variation. They are also sensitive to patient deformation, and not to rigid misalignment alone: during CSI, local reproducibility of the spinal column is limited by inter- and intrafraction changes in spinal curvature that cannot be removed by rigid whole-body alignment, so that clinically relevant residual local misalignment persists even after image-guided correction at the reference point [26]. Any change of treatment posture, including a change from supine to upright, must therefore be evaluated for its effect on segmental spinal alignment and on field-junction dosimetry, and not only for its effect on global setup accuracy.

Anesthesia use in pediatric radiotherapy declines strongly with age. Most children under four require sedation for radiotherapy, while dependence falls across early childhood and becomes less common in adolescence [23]. This age relationship is clinically important because it identifies a large intermediate group of school-aged children for whom treatment may be possible either with or without anesthesia, depending on the environment, preparation, and setup demands.

Immobilization requirements differ markedly across treatment sites and modify this age relationship. Children treated for CNS, head and neck or skull-base targets generally require a thermoplastic mask, increasingly in an open-face configuration; the mask adds a step that many young children find difficult to tolerate and it constrains direct access to the face and airway, so that mask acceptance rather than the ability to keep still is often the factor determining whether anesthesia can be avoided [[24], [27]]. Conversely, extracranial sites treated without a mask, for example extremity and trunk sarcomas, flank irradiation for Wilms tumor, or total body irradiation, place less demand on facial immobilization but greater demand on sustained whole-body stillness and, for thoracoabdominal targets, on respiratory reproducibility [28]. The two situations pose different problems in the upright posture. Mask-based sites require an upright interface that simultaneously preserves immobilization performance, airway visibility and rapid-release capability, and that can be indexed reproducibly to the backrest and headrest rather than to a couch top. Non-mask sites require postural supports that maintain trunk, pelvic and limb stability without the anchoring that a couch provides, and are likely to be more sensitive to fatigue and postural drift during longer fractions. These two categories should therefore be commissioned and evaluated separately rather than treated as a single pediatric cohort.

Modern pediatric radiotherapy increasingly incorporates proton therapy and highly conformal photon techniques with the aim of improving target coverage while reducing dose to developing normal tissue and thereby limiting late toxicity [[29], [30], [31]]. Although these approaches may improve long-term outcomes, they can also increase setup complexity and treatment time. Without child-focused preparation and support, this may reinforce reliance on anesthesia. Conversely, structured behavioral conditioning, audiovisual immersion, and individualized, child-centered setup strategies may help reduce anesthesia use in school-aged children while improving compliance and reducing stress [[32], [33]].

Reducing repeated anesthesia may shorten appointments, reduce fasting, family disruption and stress, and lessen pressure on anesthesia staffing and recovery capacity. It may also reduce exposure to the risks intrinsic to anesthesia itself: although serious events are infrequent in experienced pediatric radiotherapy services, airway and respiratory complications, aspiration, cardiovascular instability, drug-related events and vascular access complications are all reported, and cumulative exposure across a multiweek fractionated course is a legitimate concern [34]. Prospective resource data support the operational argument: in the DEGRO-QUIRO multicentre analysis of pediatric and adolescent radiotherapy, treatment delivered under general anesthesia required more staff and longer personnel time than treatment without anesthesia, principally through longer room occupancy [35]. In resource-limited centers the binding constraint is frequently anesthetic staffing, recovery space and the availability of dedicated pediatric anesthesia sessions rather than linear accelerator capacity; avoiding anesthesia in even a subset of children could therefore release treatment slots, reduce the need for an anesthetic team and recovery area to be available on every treatment day, shorten waiting times, and allow more children to be treated within an existing service. These effects are plausible but at present unquantified, and should be measured prospectively rather than assumed.

Taken together, these data identify a key target group for upright pediatric radiotherapy developments: children old enough to potentially cooperate when appropriately supported but who might otherwise receive repeated anesthesia under conventional workflows. Upright positioning is unlikely to eliminate anesthesia for all pediatric patients, but it may expand the proportion treated awake.

## Anesthesia and safety considerations in upright pediatric radiotherapy

In many centers, anesthesia for supine pediatric radiotherapy begins with induction on the procedure couch, followed by maintenance with spontaneous ventilation, supplemental oxygen, and propofol-based sedation, with continuous remote monitoring and supervised recovery [36]. Upright radiotherapy does not alter the basic principles of pediatric anesthesia, but it introduces specific issues relating to airway management, hemodynamics, positioning, and emergency access.

Airway management is central to safe upright pediatric radiotherapy. Although soft-tissue airway obstruction during spontaneous ventilation can occur in any sedated pediatric patient, upright treatment introduces specific posture- and device-related risks: seated positioning, head or thoracic supports, and immobilization devices may promote chin flexion, neck malalignment, or inadequate airway support, potentially causing obstruction before hypoventilation becomes evident [36]. Final positioning must preserve airway patency, allow continuous capnography and visual monitoring, and provide immediate access for airway intervention. This is particularly important to consider in upright systems incorporating headrests, thoracic supports, masks, or forward-leaning positions.

Positioning also changes pressure distribution and mechanical support requirements. Inadequate physical support may increase the risk of pressure injury, nerve compression, or instability during induction and treatment. Appropriate padding, neutral limb alignment, and full physical support are therefore required before induction and throughout the procedure.

Hemodynamic management must account for hydrostatic effects in the upright child. Blood pressure (BP) measured at the arm may overestimate perfusion pressure at brain level when the patient is seated. Approximately 2 mmHg per inch, or 1 mmHg per 1.25 cm, should be subtracted to estimate cerebral perfusion pressure at the brainstem [37]. Upright positioning may also reduce venous return and increase the likelihood of vasoactive support. Close BP trending and awareness of hydrostatic correction are therefore essential.

Emergency procedures must be designed into the system from the outset. During beam delivery, the anesthesiologist may be physically separated from the patient, so rapid response must be factored into equipment designs and workflows must be rehearsed. Upright systems should permit immediate beam cessation, rapid release of immobilization, and immediate access to the head and airway. Depending on the platform, the patient should either be reclined quickly to supine or removed rapidly from the device for airway management. Because upright platforms are frequently promoted as compact, space-efficient solutions, it should be stated explicitly that a small device footprint must not be achieved at the expense of the free space around the patient. The room layout must allow the anesthesia team unobstructed access to the head and airway from more than one side, permit a trolley or couch to be positioned immediately adjacent to the chair, and provide sufficient clearance for the chair to recline fully or for the child to be lifted clear of the device and laid flat without other equipment having to be moved first. Minimum clearance envelopes, door widths, transfer routes and the position of anesthesia and monitoring equipment should therefore be specified at the room-planning stage and verified during acceptance testing, rather than discovered at the first emergency. These procedures should be standardized, rehearsed and validated before clinical use.

The anticipated hazards are not fundamentally unfamiliar (see Table 1); similar airway, access, monitoring, and positioning concerns are already managed in pediatric anesthesia in other upright procedures [[7], [27], [28], [36], [37], [38]]. Table 1 therefore distinguishes hazards that are already encountered in supine pediatric radiotherapy from those that are amplified by, or specific to, the upright posture, so that departments can separate what is genuinely new from what requires extension of existing practice. The challenge is to adapt established principles reliably to a radiotherapy environment that may include immobilization devices, limited access during beam delivery, and platforms not originally designed for anesthetized seated patients. Upright pediatric radiotherapy should therefore be developed as a safety-led innovation, with emergency access and airway visibility treated as primary design constraints.

**Table 1:** Envisioned hazards, their relevance to current supine practice, and mitigations for upright anesthesia in pediatric radiotherapy.

Phase/Domain	Hazard in the upright setting	Relevance	Underlying Risk	Mitigation Strategy
**Inducing anesthesia**	Induction performed on the treatment chair or upright positioning system	Upright-specific	Patient instability, forward or lateral collapse, loss of airway alignment	Ensure full physical support prior to induction; use headrests, supportive straps and lateral supports; confirm airway alignment before loss of consciousness
**Airway positioning**	Chin flexion and neck malalignment due to seated posture and immobilization devices	Common to supine RT, but amplified upright	Soft tissue airway obstruction, hypoventilation, apnea	Elevate chin and gently extend neck; consider supportive straps, thermoplastic or open-face masks to hold head and neck alignment, verifying that they neither obscure the face nor delay release; verify airway patency after final positioning; continuous capnography and visual chest excursion monitoring
**Natural airway maintenance**	Spontaneous ventilation without a secured airway in the upright position	Common to supine RT	Rapid obstruction before hypoventilation is detected	Vigilant monitoring; conservative anesthetic depth; ready access to airway adjuncts (oral airway, face mask, laryngeal mask airway [LMA], endotracheal tube [ETT])
**Remote monitoring**	Anesthesiologist physically separated from the patient during beam delivery	Common to supine RT	Delayed response to airway or cardiovascular compromise	Continuous cardiorespiratory and capnography monitoring; immediate access protocols; clear line of sight where possible
**Hemodynamic measurement**	Blood pressure (BP) cuff positioned below the level of the brainstem	Upright-specific	Overestimation of cerebral perfusion pressure	Apply hydrostatic correction (approximately 2 mmHg per inch height difference); target higher cuff pressures as appropriate
**Upright physiology**	Reduced venous return and postural hypotension	Upright-specific	Cerebral hypoperfusion, need for vasoactive support	Close BP trending; early vasopressor use when indicated; avoid excessive anesthetic depth
**Pressure points**	Concentrated load on chin, jaw, axilla and elbows	Common to supine RT, but amplified upright (different load distribution)	Nerve injury (ulnar, brachial plexus), skin breakdown	Padding of contact areas; neutral limb positioning; frequent checks during setup
**Immobilization systems**	Restricted access to the airway and to the patient	Common to supine RT, but amplified upright	Delayed emergency intervention	Designated quick-release or access mechanisms; pre-procedure equipment checks
**Airway emergencies**	Obstruction or apnea in an immobilized upright patient	Common to supine RT, but amplified upright	Inability to ventilate rapidly	Standardized emergency sequence: stop beam → release immobilization → airway access → reposition or remove patient
**System limitations**	Some upright platforms may not recline rapidly, or may require release from immobilization before full airway access is possible	Upright-specific	Delayed transition to a supine or rescue position and delayed access for effective airway management	Confirm platform recline and removal capabilities before treatment; rehearse emergency release and patient transfer; ensure immediate beam stop, rapid immobilization release, and a predefined airway rescue workflow
**Room layout and access space**	Compact upright platform installed in a room with insufficient clearance around the patient, or with anesthesia and monitoring equipment obstructing access	Upright-specific	Inability to reach the head from more than one side, to bring a trolley alongside, or to lay the child flat without first moving other equipment; delayed airway rescue despite a functioning quick-release mechanism	Define a minimum clearance envelope at the room-planning stage; ensure access to the head from more than one side; keep a trolley or couch immediately adjacent during anesthetized fractions; verify door widths and transfer route; include room layout in acceptance testing and in emergency rehearsal
**Emergence and transport**	Recovery while still sedated after upright exposure	Common to supine RT, but amplified upright	Airway compromise during movement	Maintain close monitoring during transfer; ensure airway patency before handover

## Audiovisual distraction and behavioral support

Audiovisual distraction and immersive technologies are increasingly used in pediatric radiotherapy to reduce anxiety, improve cooperation, and decrease sedation requirements [39], [40], [41], [42], [43], [44]. These approaches are typically combined with age-appropriate preparation, play-based rehearsal, mock simulation, and psychological support [45]. Their relevance to upright radiotherapy is substantial, because awake treatment depends on the child remaining calm and engaged during repeated sessions.

**Fig. 1:**
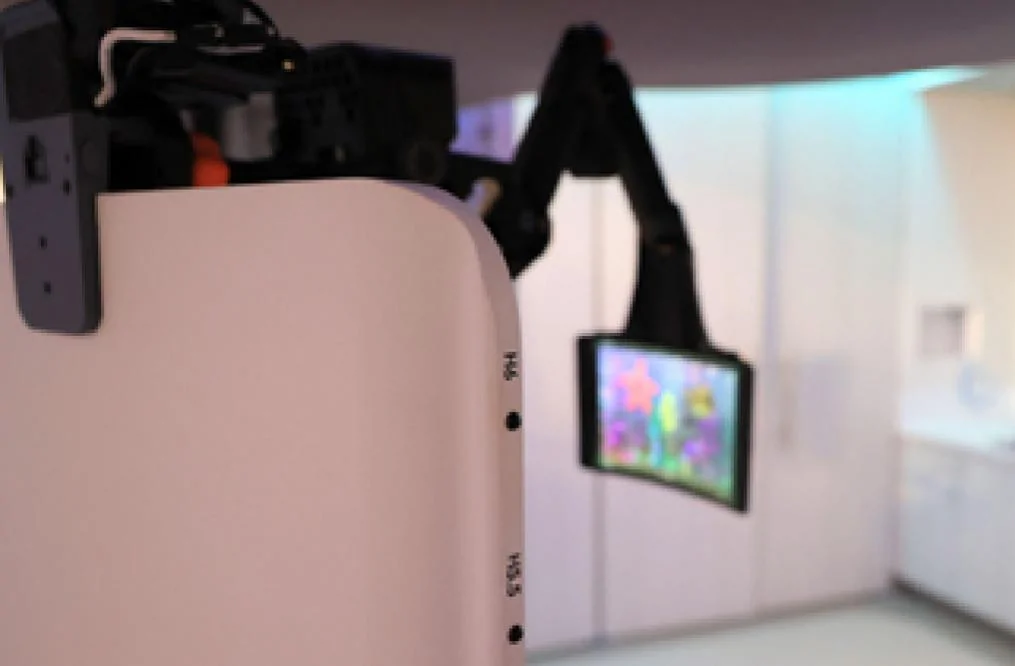
Image of AVATAR system attached to an upright patient positioning system backrest.

Systems such as the Audiovisual-Assisted Therapeutic Ambience in Radiotherapy (AVATAR) platform, developed at Stanford University, have shown feasibility in conventional supine workflows and provide a useful model for upright adaptation [41], [42], [43], [44]. In upright treatment, the display system could be mounted to the backrest or another structural component of the positioning platform, provided it does not interfere with imaging or treatment beams (see Fig. 1). The screen, or a mirror-based alternative, should remain within the patient's natural line of sight. Dosimetric studies suggest that appropriately designed systems such as AVATAR can remain in place with minimal effect in photon radiotherapy [43]; for proton radiotherapy, it is likely that beam angles would need to be carefully selected to avoid such equipment.

Two-way communication should also be integrated into upright treatment environments. As seated patients retain greater visual orientation to the room than fully supine patients, upright positioning may further support reassurance and engagement through direct communication with staff and caregivers. For some children, this may improve control and reduce treatment-related distress.

Audiovisual support is not merely an optional add-on. In pediatric upright radiotherapy, it is likely to be a core component of the treatment environment, especially for children treated awake.

## Clinical and design requirements for upright pediatric systems

Reducing distress is a central requirement. Upright systems should be ergonomic and adaptable across a wide pediatric age range, with adjustable support for the head, thorax, abdomen, pelvis, and lower limbs. Adjustable upper-limb supports are equally necessary: both arms-up and arms-along-the-body configurations will be required depending on treatment site and beam access, and in adults the choice of arm position has been shown to alter beam access, field geometry and set-up comfort in the upright posture [46]. In children, upper-limb supports must additionally scale across a wide range of limb lengths, maintain neutral shoulder and elbow alignment to avoid brachial plexus or ulnar stretch, and be reproducible from day to day without requiring sustained active cooperation. Non-invasive immobilization options, including thermoplastic masks, vacuum cushions, and open-face interfaces, should provide stability while remaining tolerable. Both forward-leaning and backward-leaning configurations may be useful depending on treatment site, age, and whether anesthesia is required.

Imaging compatibility is essential. Upright positioning systems should support fan-beam CT simulation in the treatment posture and, ideally, integration with surface guidance and orthogonal kV imaging adapted for upright geometries. Compatibility with surface-guided radiotherapy (SGRT) systems is particularly relevant, as SGRT is increasingly standard practice in pediatric departments for intrafraction motion monitoring without additional imaging dose, and optical surface tracking has already been used to quantify both inter- and intrafraction setup accuracy in the upright posture across different immobilization configurations [47]. Day-to-day setup reproducibility and within-fraction stability should both remain within clinically acceptable tolerances, and the two must be verified separately: a configuration that is reproducible between fractions may still permit progressive postural drift within a fraction, whereas a very rigid configuration may be stable but poorly reproducible if it is not tolerated by the child. Early adult data in head and neck patients suggest that upright residual setup errors can be comparable to supine values [18], but equivalent pediatric data do not yet exist. Deformable registration between supine MRI and upright CT will be an important area of development. Machine-specific quality assurance protocols for upright systems will also require development and prospective validation before clinical commissioning can be completed. Because many suitable pediatric treatment sites are likely to be above the diaphragm, these requirements are especially relevant for the head, neck, and thoracic regions.

Workflow efficiency is also important. Upright systems should support short, coordinated treatment sessions with reliable ingress, positioning, verification, and egress. The radiation therapist plays a central role in upright workflow coordination, including daily setup verification, image guidance interpretation, immobilization management, and emergency response readiness; these requirements must be addressed in departmental training and role definition before clinical implementation. When anesthesia is required, the system must support rapid airway access, continuous visibility of the patient and monitors, and immediate repositioning or removal in an emergency.

Treatment planning for upright patients will require validation of beam geometry, gravity-related anatomical changes and their dosimetric impact for treatment planning. Upright postures may alter the relative positions of targets and organs at risk, creating both advantages and disadvantages and new planning challenges. These effects are likely to vary by site and must be characterized systematically rather than assumed.

Three main pediatric user groups can be considered when specifying upright systems for pediatric use, and the design implications differ between them: (1) adolescents and larger children who already fit adult-oriented upright radiotherapy systems; (2) children treated awake who are too small for adult systems; and (3) children requiring anesthesia who are too small for adult systems. We are not proposing a separate new device for each group. For group 1, no new device is required: the need is for pediatric-appropriate accessories, age-adapted preparation and audiovisual support, and prospective validation of existing adult platforms in this younger population, which makes this the group in which clinical evaluation could begin soonest. For group 2, scaled-down supports, smaller adjustment ranges and child-appropriate ergonomics are required, but the safety envelope remains that of an awake, cooperating patient. Group 3 is where genuinely new or substantially modified hardware is most likely to be necessary, and it places the greatest demands on safety and emergency access. Across all groups, devices should allow immediate access to the head, torso, and airway; rapid release of immobilization; and fast evacuation from the imaging or treatment environment if required [[27], [48], [49]]. Airway visibility and access must remain unobstructed, particularly in anesthetized patients and in forward-leaning configurations [[27], [28]]. Monitoring lines and vital-sign equipment must also remain visible and secure [50].

In addition, supports should be radiotranslucent if they fall within likely beam paths, compatible with existing immobilization systems where relevant, and designed to accommodate integrated distraction technologies [51], [52], [53]. Compatibility with established thermoplastic and vacuum-based systems is especially important, as it may lower adoption barriers and reduce the need for new departmental infrastructure.

## Workflow considerations

Simulation and treatment workflows for upright pediatric radiotherapy should mirror conventional pathways while incorporating posture-specific setup, immobilization, reproducibility and stability checks, and imaging in the intended treatment posture. Daily treatment should reproduce the simulation position using stored device settings, alignment tools, and upright image guidance. The core workflow requirements are day-to-day reproducibility, within-fraction stability, efficient patient ingress and egress, and compatibility with routine verification and correction processes.

The radiation therapist team is central to workflow implementation. In the upright setting, their responsibilities will include: confirmation of correct device configuration and stored setup parameters at simulation and each treatment fraction; daily positioning verification using upright image guidance; coordination with the anesthesia team during induction, monitoring, and recovery for sedated patients; real-time communication with awake children via two-way audiovisual systems; and initiation of emergency protocols if patient safety is compromised. These responsibilities extend beyond conventional supine radiation therapy workflows and will require site-specific training, protocol development, and role clarification prior to clinical introduction. Workflow rehearsal, including emergency scenarios, should be conducted with the full multidisciplinary team before treating the first patient.

Potential workflow advantages of upright positioning, including easier patient ingress and egress, reduced transfer requirements, and shorter setup or repositioning times, should be evaluated prospectively rather than assumed. Adult data from upright pelvic radiotherapy suggest feasible positioning times and favorable patient-reported comfort [54], but these findings will need dedicated validation in pediatric workflows, where immobilization, anxiety management, audiovisual support, and anesthesia may substantially alter timing and staffing requirements.

## Evidence development for clinical implementation

**Fig. 2:**
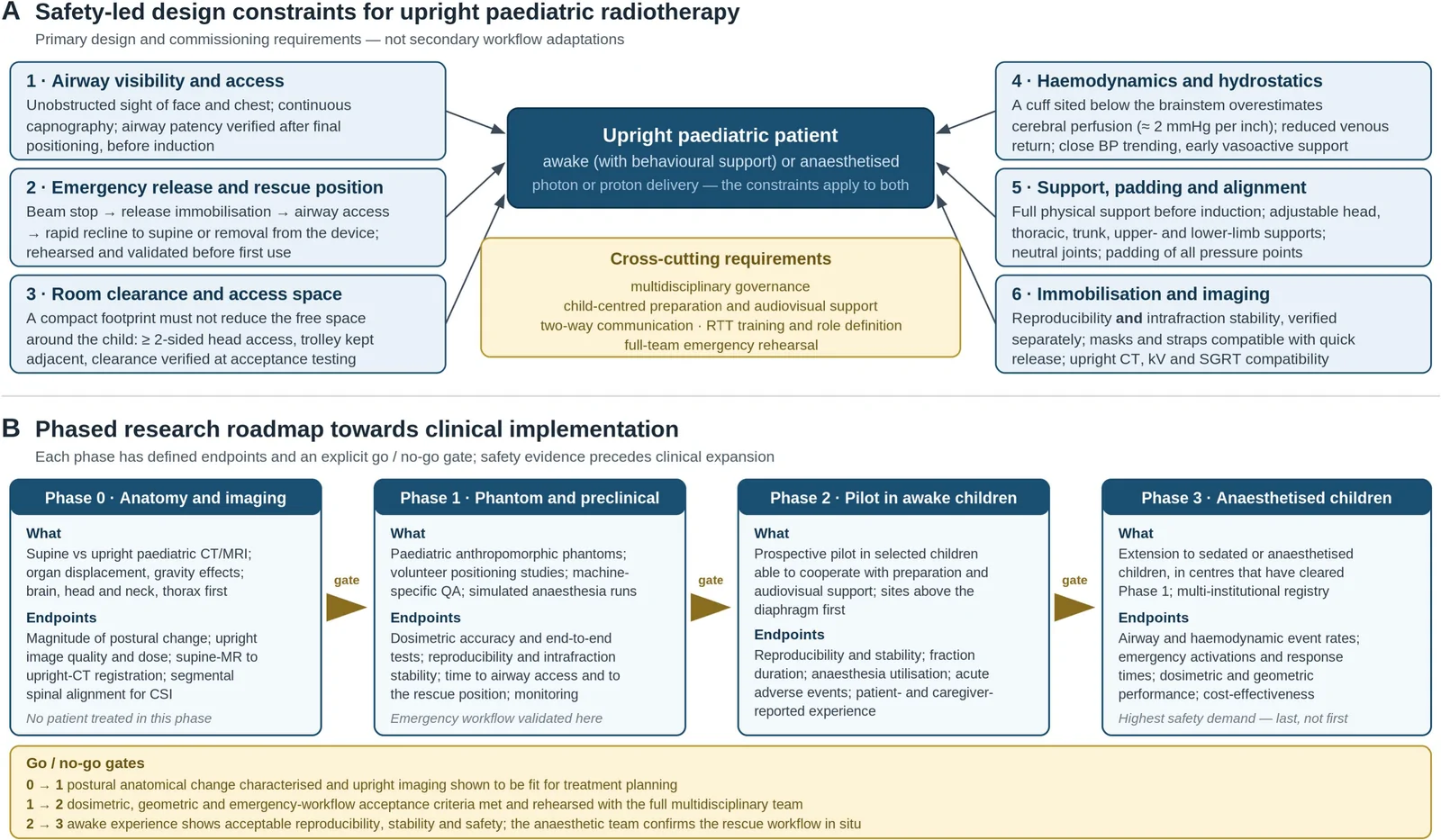
Framework for the safe translation of upright pediatric radiotherapy. (A) Safety-led design constraints that should be treated as primary design and commissioning requirements for upright systems intended for pediatric use, together with the cross-cutting organizational requirements that apply regardless of platform. (B) Phased research roadmap, in which each phase has defined endpoints and an explicit go/no-go gate, so that safety evidence precedes clinical expansion and treatment of anesthetized children is approached last rather than first. BP, blood pressure; CSI, craniospinal irradiation; CT, computed tomography; kV, kilovoltage; MR(I), magnetic resonance (imaging); QA, quality assurance; RTT, radiation therapist; SGRT, surface-guided radiotherapy.

Fig. 2 summarizes the safety domains that must be controlled and the sequence of evidence required before and during clinical introduction. Initial anatomical and dosimetric studies should compare pediatric anatomy in supine and upright positions using CT and MRI, focusing on organ displacement, reproducibility, stability, and gravity-related changes relevant to treatment planning [19], [55], [56]. Because pediatric indications are commonly above the diaphragm, early studies should prioritize the brain, head and neck, and thorax.

Pediatric anthropomorphic phantoms and preclinical motion studies will be needed to validate image quality, imaging dose, dosimetric accuracy, setup reproducibility, within-fraction stability, and emergency workflows in the upright setting. These should include anesthesia scenarios, access times, clearance around the patient, and monitoring arrangements.

Pilot clinical studies should then assess whether upright treatment improves acceptability, cooperation, setup efficiency, and anesthesia avoidance for children and families. Such studies should use standardized endpoints, including setup reproducibility and within-fraction stability, fraction duration, anesthesia utilization, acute adverse events, and patient- and caregiver-reported outcomes using validated pediatric measures of distress, cooperation, and procedural experience.

Technology development should proceed in parallel. Priorities include upright image guidance, treatment planning methods, and support for forward- and backward-leaning chair geometries. Engagement with clinicians, physicists, anesthesiologists, child-life specialists, patients, and families will be essential during iterative system design.

Implementation research should also address assent and consent pathways, regulatory approval, cost-effectiveness, and workflow integration, particularly as upright-only treatment environments emerge. Early implementation studies will be important to ensure that technical enthusiasm does not outpace safety evidence.

## Conclusions

Upright pediatric radiotherapy is historically grounded and increasingly plausible with modern imaging, immobilization, anesthesia, and behavioral support strategies. It may bring advantages in terms of reduced distress, improved cooperation, and lower dependence on anesthesia in selected children, alongside possible anatomical or dosimetric benefits for some patients.

However, the concept should be advanced cautiously. Near-term priorities are safety-focused system design, validation of upright imaging and immobilization, and prospective studies evaluating setup reproducibility and within-fraction stability, workflow efficiency, anesthesia utilization, and patient-family experience. Multi-institutional collaboration and outcome reporting will be essential.

Upright treatment is unlikely to replace conventional supine radiotherapy for all pediatric patients. Its value will depend on identifying the patient groups, treatment sites, and clinical workflows in which it offers benefit. If developed within a rigorous safety and evidence framework, upright pediatric radiotherapy merits prospective evaluation as a child-centered extension of modern radiotherapy practice.

## Declaration of generative AI and AI-assisted technologies.

The framework, ideas and content behind this manuscript are the authors' own. During manuscript preparation, ChatGPT-5 Plus was used to edit and refine the text. Privacy controls were implemented such that content provided to ChatGPT was not used to train the AI model. Edited text was carefully verified by the authors to ensure accuracy; the authors take full responsibility for the content of the final publication.

## Task group

This manuscript was prepared by the Upright Radiotherapy Pediatric Task Group, an international, multiprofessional working group whose origin, membership, sponsorship status and terms of reference are described at the end of the Introduction.

## CRediT authorship contribution statement

**Lorenzo Placidi:** Writing – review & editing, Writing – original draft, Validation, Methodology, Data curation, Conceptualization. **Kathryn Yip:** Writing – review & editing, Writing – original draft, Supervision, Methodology, Data curation, Conceptualization. **Yat Man Tsang:** Writing – review & editing, Writing – original draft, Validation, Methodology, Formal analysis, Data curation. **Samuel Rodriguez:** Writing – review & editing, Writing – original draft, Methodology, Data curation. **Lawrie Skinner:** Writing – review & editing, Writing – original draft, Validation, Methodology, Formal analysis, Data curation, Conceptualization. **Tracy Underwood:** Writing – review & editing, Writing – original draft, Methodology, Data curation, Conceptualization. **Niek Schreuder:** Writing – review & editing, Writing – original draft, Validation, Supervision, Project administration, Methodology, Formal analysis, Data curation, Conceptualization.

## Funding

This research did not receive any specific grant from funding agencies in the public, commercial, or not-for-profit sectors.

## Declaration of competing interest

The authors declare the following financial interests/personal relationships which may be considered as potential competing interests: Kate Yip, Tracy Underwood and Niek Schreuder are employed by Leo Cancer Care, a company developing equipment for upright radiotherapy. All other authors declare no known competing financial interests or personal relationships that could have appeared to influence the work reported in this manuscript. Management of this conflict during preparation of the manuscript is described at the end of the Introduction and summarised in the Conflict of Interest statement: the scope, structure and principal conclusions were agreed by the authors without a commercial affiliation before drafting began; no passage recommending clinical adoption, safety requirements, evidence thresholds or research priorities was written or finally edited by an author with a commercial interest; no specific commercial system is named, compared or endorsed, and device-related statements are supported by peer-reviewed publications only; and the corresponding author, who has no commercial affiliation, retained final editorial control over content and wording.
